# Experimental research on the spontaneous combustion of Yangquan coal induced by electrochemical oxidation of pyrite

**DOI:** 10.1038/s41598-022-04864-1

**Published:** 2022-01-18

**Authors:** Xun Zhang, Bing Lu, Xiang Fu, Ling Qiao, Jiren Wang, Lijie Wang, Cong Ding, Dameng Gao, Jing Zhang

**Affiliations:** 1 College of Mining Engineering, Liaoning Technical University, Fuxin, 123000 Liaoning China; 2 College of Safety and Emergency Management Engineering, Taiyuan University of Technology, Taiyuan, 030000 Shanxi China; 3 College of Safety Science and Engineering, Liaoning Technical University, Fuxin, 123000 Liaoning China; 4 China Coal Technology and Engineering Group Nanjing Design and Research Institute Co., Ltd., Nanjing, 210031 Jiangsu China

**Keywords:** Fossil fuels, Electrocatalysis

## Abstract

The 15# coal seam of Yangmei No.5 Mine, which produces anthracite, which is the least prone to spontaneous combustion, has a serious hidden danger of spontaneous combustion due to the high sulfur content in the coal. Based on the better conductivity of anthracite, we designed an electrolysis experiment to accelerate the electrochemical oxidation of pyrite in coal. Through experiments and analysis of thermodynamic characteristic parameters, it is obtained that the electrochemical oxidation of pyrite and its main products Fe^3+^ and Fe^2+^ have a coupled catalytic effect on the spontaneous combustion of high-sulfur coal in Yangquan. Combined with the FTIR test and analysis, it is found that the electrochemical process causes spatial polarization in the coal, so that polar groups such as –OH undergo spatial diversion and increase the activity. Due to the high content of –OH in Yangquan anthracite, the electrochemical process has the greatest effect on promoting –OH oxidation. Fe^3+^ and Fe^2+^ act as strong oxidants and free radicals to promote the –CH_2_– reaction to generate C=O and promote the generation of CO. This research provides a new direction for the exploration of the spontaneous combustion mechanism of high-sulfur anthracite.

## Introduction

Pyrite is one of the main internal factors affecting the spontaneous combustion of coal. When the pyrite content is high, even if the anthracite with a high degree of metamorphism is mined, the spontaneous combustion period in the mined-out areas will be greatly shortened. Therefore, the research on the mechanism of pyrite-catalyzed coal spontaneous combustion is particularly important. The oxidation forms of pyrite in coal mainly include chemical oxidation, microbial oxidation and electrochemical oxidation. Most scholars have studied that pyrite promotes coal spontaneous combustion from the perspective of its chemical oxidation and heat release. It is believed that the oxidation reaction of pyrite underground is the same as the chemical reaction that occurs under the experimental conditions. The heat released during the chemical reaction promotes the temperature increase of coal is the main mechanism that promotes the spontaneous combustion of coal^1–3^. The three types of exothermic reactions are as follows.

1 $${\text{2FeS}}_{{2}} + {\text{2H}}_{{2}} {\text{O}} + {\text{7O}}_{{2}} \longrightarrow {\text{2FeSO}}_{{4}} + {\text{2H}}_{{2}} {\text{SO}}_{{4}}$$

2 $${\text{12FeSO}}_{{4}} + {\text{6H}}_{{2}} {\text{O}} + {\text{3O}}_{{2}} \longrightarrow{\text{4Fe}}_{{2}} ({\text{SO}}_{{4}})_{{3}} + {\text{4Fe}}\left( {{\text{OH}}} \right)_{{3}}$$

3 $${\text{FeS}}_{{2}} + {\text{Fe}}_{{2}} ({\text{SO}}_{{4}})_{{3}} + {\text{3O}}_{{2}} + {\text{2H}}_{{2}} {\text{O}}\longrightarrow {\text{3FeSO}}_{{4}} + {\text{2H}}_{{2}} {\text{SO}}_{{4}}$$

Solverman^4^ proposed the hypothesis of bacterial leaching for the first time. Hunte et al.^5^ used Thiobacillus ferrooxidans to remove pyrite from coal, and then analyzed the characteristics of coal. It is believed that the removal of pyrite by bacteria can reduce the ash and volatile content in coal. Zhang et al.^6^ through XRD and SEM/TEM experiments believed that the oxidation of T.f bacteria on the coal surface is a relatively mild oxidation of pitting corrosion. This shows that the bacterial oxidation form of pyrite has less and slower impact on coal spontaneous combustion.

For the electrochemical oxidation form of pyrite, scholars at home and abroad have done a lot of research work^7–9^. Wu et al.^10–12^ believed that pyrite can react electrochemically with the contact surface of water and humid environment, and the potential difference, electrolyte and oxidant and electron channels are the basic conditions for electrochemical oxidation of pyrite. Kelsall^13^ believes that the electrochemical oxidation of pyrite is a complicated series and parallel reaction step, but the reaction eventually produces Fe^2+^/Fe^3+^ and S/S_2_O_3_^2−^/SO_4_^2−^. Tu^14,15^ found that in a humid environment, the main oxidation equation for electrochemical reaction of pyrite is:

$$\begin{aligned} & {\text{positive electrodes: FeS}}_{2} + 8{\text{H}}_{2} {\text{O}} = {\text{Fe}}^{2 + } + 2{\text{e}}^{ - } + 2{\text{SO}}_{4}^{2 - } + 16{\text{H}}^{ + } + 14{\text{e}}^{ - } \\ & {\text{negative electrode: O}}_{2} + 4{\text{H}}^{ + } + 4{\text{e}}^{ - } = 2{\text{H}}_{2} {\text{O}},\,{\text{Fe}}^{3+} + {\text{e}}^{ - } = {\text{Fe}}^{2+} \\ \end{aligned}$$

Xu and Du^16–18^ believed that as believed that as the degree of metamorphism increased, the aromatic structure and the degree of polycondensation in coal increased, and the conductivity of coal increased, which provides conditions for the electrochemical oxidation of pyrite in Yangquan anthracite. Qiu et al.^19^ believed that the surface of high-sulfur anthracite in a humid environment provides the oxidant and oxidizing environment required for the cathode reaction. Under the action of desulfurization bacteria, the pyrite embedded in the depth of the coal provides the reducing agent and reducing environment required for the anode reaction, thereby forming a natural redox electric field in the high-sulfur coal. In a humid environment, the pyrite exposed to the outside of the coal will form an electrochemical reaction on the surface to accelerate oxidation. Ogunsola et al.^20^ believes that the acidic conditions in coal can enhance the electrochemical oxidation potential of pyrite.

In summary, the electrochemical oxidation of pyrite is one of the main oxidation forms of pyrite in coal. However, there is currently no research to systematically analyze the influence of the electrochemical oxidation of pyrite on the spontaneous combustion of coal. The coal quality of No.15 coal in Yangquan No.5 Mine is anthracite, which is not easy to combust. As its pyrite content averages 2.7%, the spontaneous combustion time of the coal seam is greatly shortened. Based on the strong conductivity of anthracite, an acidic electrolytic cell with HCl as the electrolyte is designed to promote the electrochemical oxidation of pyrite in coal. Thermodynamic experiments are used to analyze the spontaneous combustion characteristics of electrolyzed coal and coal samples added with pyrite oxidation products. Then through the FTIR test to analyze the changes in the content of active groups in the coal, the effect of electrochemical oxidation of pyrite on the spontaneous combustion of Yangquan anthracite is obtained.

## Experiments and methods

### Preparation of coal samples

This paper selects the high and low sulfur anthracite coal from the adjacent mining area of No.15 coal seam in Yangquan No.5 Mine as the experimental coal sample. Under the air condition, use YJ-500A ore crusher to crush the coal. The particle size of the experimental coal sample is selected from 100 to 250 mesh. The labeled coal sample H_0_ is a high-sulfur coal. The detection shows that the inorganic sulfur content of the H_0_ high-sulfur coal accounts for 93% of the total sulfur content, and the low-sulfur coal sample L_0_ is used as a control group. Industrial analysis is shown in Table 1.

**Table 1:** Technical parameters of coal samples (%).

Sample	Moisture	Ash	Volatile matter	Total sulfur	Sulfate sulfur	Inorganic sulfur	Organic sulfur
H_0_	2.70	11.33	10.5	3.5	0.05	3.3	0.15
L_0_	1.89	40.75	12.5	0.70	0.02	0.59	0.09

### Electrolytic experiment

In order to accelerate the electrochemical oxidation of pyrite in coal, a diaphragmless electrolysis experiment was prepared by ourselves. The experimental steps are as follows: ① Add 0.75 mol/L HCl solution as electrolyte solution to the self-made diaphragmless electrolytic cell, add coal sample to make a coal slurry mixture of 0.025 g/mL, and stir it with an electric stirrer to form a uniform coal water slurry. ② Electrolyze for 3 h and 8 h at a constant current with a current density of 0.069A/cm^2^ using graphite electrodes. ③ After the electrolysis, the coal slurry is placed in a Buchner funnel with qualitative filter paper for suction filtration, and the electrolyzed coal is repeatedly washed with deionized water. ④ Detect whether the filtrate contains Cl^−^ with AgNO_3_ reagent until there is no white precipitate in the filtrate.

### TG-DSC experiment

The coal sample was dried in a vacuum drying oven at 70 °C for 24 h. Under a specific atmosphere (gas atmosphere is 50 ml/min air flow, weight loss heating rate 10 °C/min, test temperature range is 25–700 °C), the thermal weight loss behavior of coal samples is analyzed using the German Netzsch STA449F3 instrument.

### Adiabatic oxidation and index gas experiment

**Figure 1:**
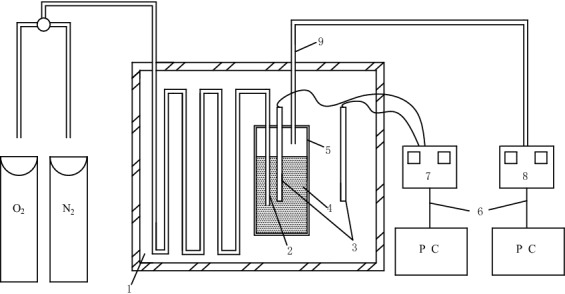
Experimental principle of adiabatic oxidation. 1. Insulated box, 2. Inlet port, 3. Temperature sensor, 4. Coal, 5. Adiabatic oxidation bottle, 6. Data collection, 7. Temperature controller, 8. Gas chromatograph, 9. Escape port.

According to the adiabatic oxidation experimental prediction model^21–23^, the laboratory has developed adiabatic oxidation experimental equipment by itself, and collected the CO gas generated during the coal oxidation process. The principle of the device is shown in Fig. 1. The experimental procedure is as follows: 100 g coal sample dried in a vacuum at 110 °C for 24 h is placed in an adiabatic oxidation bottle in an adiabatic furnace, and the bottle cap is tightly fastened. Keep at a constant temperature of 40 °C for 2 h in a nitrogen atmosphere. Subsequently, oxygen at a flow rate of 50 ml/min was introduced. The temperature of the adiabatic oxidation bottle and the adiabatic furnace are detected by the temperature detector, and the temperature of the two is kept as consistent as possible by the controller (the amplitude does not exceed 0.2 °C). The gas is collected regularly at the exhaust port of the oxidation bottle, and the CO content produced during the low-temperature oxidation of coal is analyzed by a meteorological chromatograph.

### FTIR analysis

The experimental instrument is Germany TENSOR27 Fourier transform infrared spectrometer. Grind the coal sample with a mortar to a size of 250 mesh or more, and then vacuum-dry it at a constant temperature of 110 °C for 4 h to remove moisture from the coal. After cooling to room temperature, the coal and KBr are fully mixed in proportion and pressed into tablets.

## Conclusion and analysis

### Results of electrolysis experiment

The high-sulfur coal H_0_ and low-sulfur coal L_0_ after electrolysis 3 h and 8 h are recorded as H_1_ and H_2_, L_1_ and L_2_. The results are shown in Table 2. After testing, after 3 h and 8 h of electrolysis, the removal rate of pyrite in H_0_ was 41% and 72%, and the removal rate of pyrite in L_0_ was 40% and 67%. This shows that the desulfurization effect of Yangquan high-sulfur coal is obvious under the electrolysis conditions. From the point of view of electrochemical reaction, the electrochemical oxidation reaction of pyrite in H_2_ proceeds more thoroughly. In addition, the ash content in coal is also removed accordingly. H_0_ and L_0_ have 3.27% and 3.19% of ash removed after 8 h of electrolysis. Since the amount of pyrite removed from the 8 h electrolysis of high-sulfur coal is 2.45%, the same amount of Fe^2+^ and Fe^3+^ as the amount of pyrite removed is added to the 8 h electrolysis coal, which is recorded as L_3_ (H_3_) and L_4_ (H_4_).

**Table 2:** Sulfur removal rate in coal after 3 h and 8 h electrolysis.

Sample		Electrolysis duration (h)	Total sulfur (%)	Sulfate sulfur (%)	Inorganic sulfur (%)	Organic sulfur (%)	Ash (%)
H_0_		0	3.5	0.05	3.30	0.15	11.33
		3	2.1	0.02	1.95	0.13	8.55
		8	1.05	0.01	0.93	0.11	8.06
L_0_		0	0.7	0.02	0.59	0.09	40.75
		3	0.42	0	0.34	0.08	39.89
		8	0.23	0	0.17	0.06	37.56

### Analysis of the influence of electrochemical oxidation of pyrite on the characteristic temperature of coal

**Figure 2:**
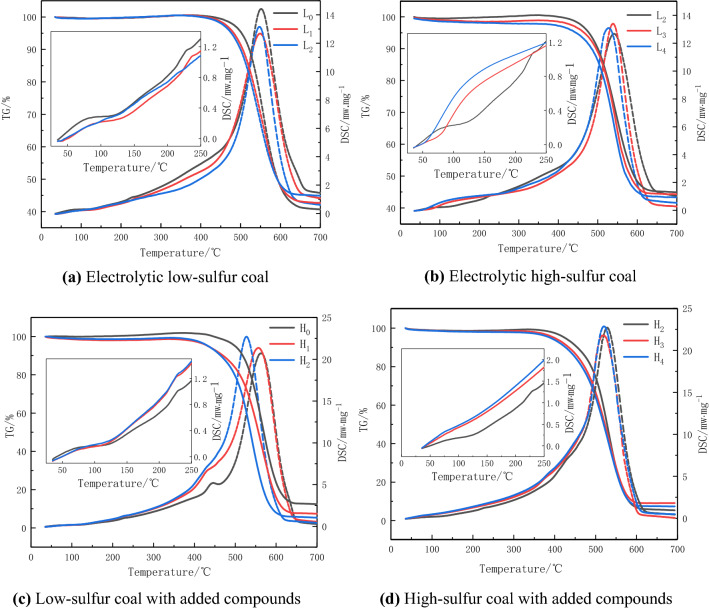
TG-DSC curves of coal samples before and after electrolysis.

This paper analyzes the influence of electrochemical oxidation of pyrite on the oxidation process of coal from the perspective of the characteristic temperature on the TG curve of the coal temperature-programming process and the low-temperature oxidation heat release on the DSC curve. The experimental results are shown in Fig. 2. The characteristic temperature is a characteristic point on the TG curve that reflects the recombination of coal and oxygen. This paper selects two characteristic temperatures, the dry cracking temperature T_1_ and the ignition temperature T_2_, to analyze the spontaneous combustion characteristics of coal. The dry cracking temperature T_1_ represents the temperature at which the weight of coal reaches a minimum before reaching the ignition temperature^24,25^. At this temperature, the reduced weight of the physically and chemically resolved gases in the coal and the increased weight of the adsorbed oxygen reach a balance. After this temperature, the oxygen absorption capacity of the coal sample is greater than the gas desorption capacity. This temperature point can reflect the reaction situation of coal oxygen under low temperature conditions. The ignition temperature point T_2_ is the temperature point at which the quality of the coal sample reaches its maximum value again as the temperature increases. After this temperature, the quality of coal drops rapidly, and the volatiles begin to burn. This temperature point can reflect the spontaneous combustion tendency of coal. The DSC curve is the heat release curve of the coal temperature program. Analyzing the heat release in the low temperature stage and the peak temperature of the combustion heat release can further obtain the influence of the electrochemical oxidation of pyrite on the spontaneous combustion of coal.

According to the TG curve in Fig. 2a, the dry cracking temperature T_1_ of low-sulfur coal L_0_ can be obtained as 118.2 °C. After 3 h and 8 h electrolysis, the dry cracking temperatures of coal samples L_1_ and L_2_ are 115.5 °C and 101.0 °C, respectively. It shows that electrolysis mainly promotes the cracking of bridge bonds, alkyl side chains and oxygen-containing functional groups in low-sulfur coal under low temperature conditions and decomposes the gas, so that the dry cracking temperature of coal samples is reduced. It can also be seen from the DSC curve that electrolysis did not promote the increase in heat release of low-sulfur coal in the low-temperature stage (25–250 °C). It shows that there is no obvious further coal-oxygen reaction in low-sulfur electrolytic coal. It can be seen from Fig. 2b that the cracking temperature of L_3_ is 212 °C, and the cracking temperature of L_4_ cannot be clearly obtained from the figure. Combined with the increase in the heat release of coal in the low temperature stage on the DSC curve, it shows that Fe^2+^ and Fe^3+^ promote the advancement of coal oxygen reaction and generate gaseous products such as CO and CO_2_ to reduce the weight of coal. It can be considered that Fe^3+^ has a greater ability to promote coal-oxygen reaction than Fe^2+^. The ignition temperature T_2_ of L_0_ is 376 °C, while the ignition temperature T_2_ of L_1_ and L_2_ are reduced to 369 °C and 357 °C, respectively. The ignition temperature T_2_ of L_3_ is reduced to 347 °C. The peak temperature of combustion exotherm of L_1_, L_2_, L_3_ and L_4_ is lower than that of L_0_. This shows that electrolysis can promote the spontaneous combustion of low-sulfur coal to a certain extent. But in comparison, Fe^3+^ and Fe^2+^ have stronger ability to promote spontaneous combustion of coal.

Figure 2c shows that for high-sulfur coal, the dry cracking temperature T_1_ of H_0_ is 115 °C, and the dry cracking temperature T_1_ of coal samples H_1_ and H_2_ after 3 h and 8 h electrolysis is 171 °C and 198 °C. On the DSC curve, the low-temperature stage (25–250 °C) of H_1_ and H_2_ emits much more heat than that of H_0_. This shows that the electrolysis of pyrite has a huge impact on the natural oxidation process of coal. While bridging bonds and alkyl side chains in coal are resolved as the temperature increases, the electrochemical oxidation of pyrite promotes the reaction of coal oxygen at low temperatures and releases gas, which in turn leads to an increase in the temperature of dry cracking of coal samples. The ignition temperature T_2_ of H_0_ is 375.9 °C, and the ignition temperature T_2_ of H_1_ and H_2_ is reduced to 346 °C and 342 °C. The temperature of the combustion exothermic peak in coal also further decreases with the increase of electrolysis time. This shows that the electrochemical oxidation of pyrite has a catalytic effect on the spontaneous combustion of coal. From Fig. 2d, the dry cracking temperature of H_3_ increased to 219.6 °C, and the ignition temperature decreased to 316 °C. There is no extreme point on the TG curve of H_4_. Combined with the heat release of the DSC curve at the low temperature stage of coal, it is believed that the influence of Fe^3+^ and Fe^2+^ on the oxidation process of electrolytic high-sulfur coal is greater than that of low-sulfur electrolytic coal.

### Analysis of pyrite electrochemical oxidation on low-temperature oxidation heat release characteristics of Yangquan anthracite

**Figure 3:**
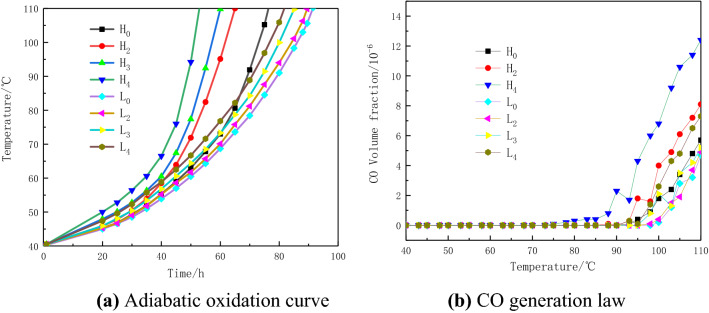
Adiabatic oxidation curve and CO generation curve of coal sample.

In order to further study the influence of the electrochemical oxidation of pyrite on the heat release of coal at low temperature, adiabatic oxidation experiment was designed to simulate the natural oxidation of coal. The CO indicator gas generated during the adiabatic oxidation process of coal is collected to assist in explaining the low-temperature oxidation of coal. In this paper, the time for the coal sample to self-heat from 40 to 110 °C, is regarded as the shortest ignition time of coal^26,27^. The experimental results are shown in Fig. 3.

It can be seen from Fig. 3 that the shortest ignition time of Yangquan high-sulfur raw coal H_0_ is 76.3 h. After the 8 h electrolysis experiment, the shortest ignition time of coal sample H_2_ was shortened to 65.3 h, which was 0.86 times that of H_0_. The shortest ignition time of coal samples H_3_ and H_4_ was shortened to 60.0 h and 52.6 h, which were 0.79 times and 0.69 times that of H_0_. The shortest ignition time of Yangquan low-sulfur coal L_0_ is 91.4 h. After the 8 h electrolysis experiment, the shortest ignition time of coal sample L_2_ was shortened to 89.7 h, which was 0.98 times that of raw coal. The shortest fire time of L_3_ and L_4_ is shortened to 85.3 h and 81.9 h, which are 0.93 times and 0.90 times that of L_0_. The analysis shows that by comparing the changes in the shortest ignition time of low-sulfur coal before and after electrolysis, high-sulfur coal exhibits stronger low-temperature oxidation ability after electrolysis. Electrolysis has a stronger promoting effect on the low-temperature oxidation of high-sulfur coal. Similarly, Fe^3+^ and Fe^2+^ promote low-temperature oxidation of high-sulfur electrolytic coal significantly stronger than low-sulfur coal. Comparing the shortest ignition time of L_3_, L_4_ and H_2_, the promotion effect of electrochemical oxidation on the low-temperature oxidation of coal is stronger than the promotion effect of iron ions in the oxidation products. Combined with the analysis of TG and DSC curves, it is obtained that the electrochemical oxidation of pyrite and its products have a coupling catalytic effect on the natural oxidation of coal. When the pyrite in coal undergoes electrochemical oxidation, its oxidation products and the form of electrochemical oxidation have a stronger promoting effect on the spontaneous combustion of coal.

Table 3 shows the situation of CO generation. Analyzing the appearance temperature and production amount of the symbol gas CO during the low-temperature oxidation process of coal can be obtained: the sequence of CO production in the experimental coal samples is basically the same as the sequence of the shortest ignition time. This shows that the electrochemical oxidation of pyrite promotes the coal-oxygen reaction to start at a lower temperature. Comparing the amount of CO produced, Fe^3+^ has a stronger ability to promote the formation of CO from coal oxygen than Fe^2+^.

**Table 3:** CO generation law under low temperature oxidation of coal.

Coal sample	H_0_	H_2_	H_3_	H_4_	L_0_	L_2_	L_3_	L_4_
Emergence temperature (℃)	93	88	86	75	100	98	97	92
The amount of production at 110 °C/ppm	5.7	8.1	8	12.4	4.7	4.9	5.2	7.3
Ratio	–	1.42	1.40	2.18	–	1.04	1.11	1.55

### FTIR experiment analysis of the mechanism of electrochemical oxidation on coal natural oxidation

**Figure 4:**
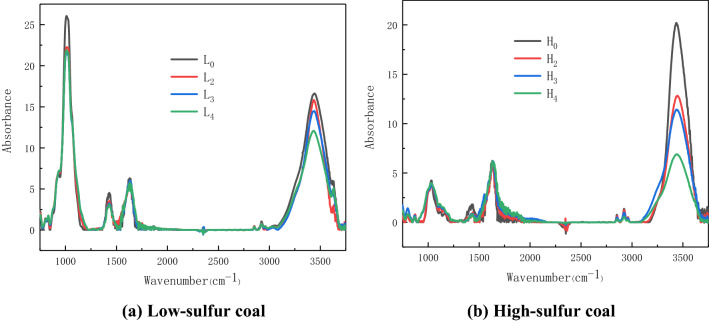
Infrared spectrum curve of coal sample.

In order to further analyze the mechanism of the electrochemical oxidation of pyrite to promote the low-temperature oxidation of coal, the coal sample at 110 °C after the adiabatic oxidation experiment was subjected to FTIR experimental analysis. The experimental results are shown in Fig. 4.

**Figure 5:**
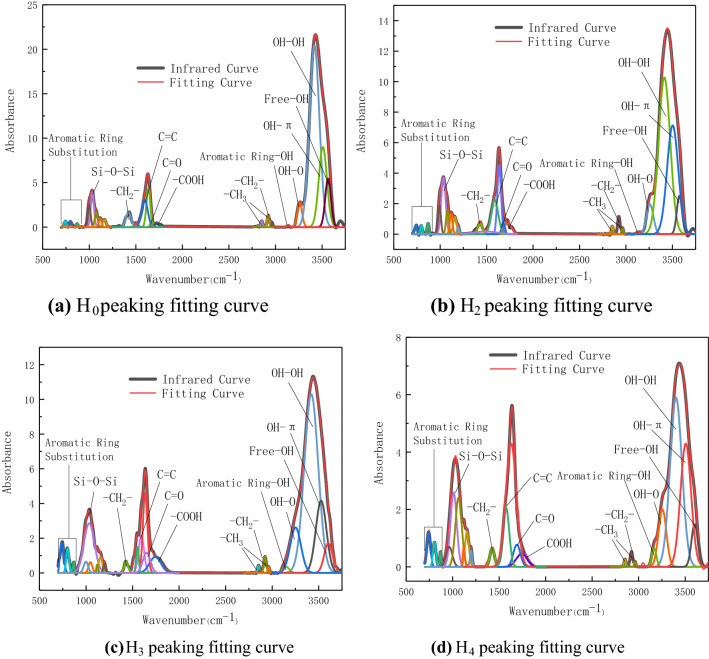
Peak fitting curve of high-sulfur coal.

From Fig. 4a, after the adiabatic oxidation experiment, the –OH absorption peak in the 3000–3600 cm^−1^ region of L_2_, L_3_, and L_4_ is significantly lower than that of L_0_. It shows that both the electrolysis process and iron ions promote the oxidation of –OH structure in coal. The acidic electrolytic cell has a greater impact on the ash content in coal. The Si–O–Si functional group at around 1020 cm^−1^ is significantly reduced. Other active structures in coal, such as alkane structure and oxygen-containing functional groups, did not change significantly. Figure 4b shows that the content of active structural aliphatic hydrocarbons (3000–2800 cm^−1^), oxygen-containing functional groups such as hydroxyl (3000–3600 cm^−1^) and C=O (1650–1700 cm^−1^) in the high-sulfur coal before and after the experiment has obvious changes. In order to further analyze the influence of pyrite electrochemical oxidation forms and products on the active groups during low-temperature oxidation of Yangquan anthracite, Peakfit software was used to peak fitting the infrared spectra of coal. Quantitative calculation and analysis of changes in active structure content in coal. The fitting result is shown in Fig. 5.

During the process of spontaneous combustion, the active groups in coal undergo oxidation reactions with lower activation energy, release heat, accumulate temperature, and then generate deep-level reactions requiring higher activation energy.

In the process of low-temperature oxidation, aliphatic hydrocarbons and oxygen-containing functional groups are the main active groups in coal, which participate in the oxidation reaction of coal in the low-temperature stage^28^. Therefore, this paper chooses C=O, –CH_2_– and –OH as the analysis objects to calculate the content changes of the three types of groups in different experimental coal samples. The results are shown in Table 4.

**Table 4:** Active functional group content in coal.

Attribute	Specimen	Band position	Width	Height	Band area	Total area of band	Proportion	Ratio
C=O	H_0_	1662	27.06	0.44	12.90	5482.04	2.35E−03	
	H_2_	1678	39.66	0.96	32.28	4246.64	7.60E−03	3.2
	H_3_	1650	94.25	1.16	117.10	4710.23	2.49E−02	10.6
	H_4_	1690	89.14	0.77	73.20	3363.91	2.18E−02	9.3
–CH_2_–	H_0_	2922	28.69	1.35	41.29	5482.04	7.53E−03	
	H_2_	2923	30.11	1.16	37.31	4246.64	8.79E−03	1.2
	H_3_	2923	32.38	0.93	32.24	4710.23	6.84E−03	0.9
	H_4_	2923	31.59	0.52	17.70	3363.91	5.26E−03	0.7
OH–OH	H_0_	3418	124.98	20.39	2712.76	5482.04	4.95E−01	
	H_2_	3413	146.53	10.27	1602.22	4246.64	3.77E−01	0.8
	H_3_	3419	176.49	10.29	1934.40	4710.23	4.11E−01	0.8
	H_4_	3401	155.54	7.89	1144.31	3363.91	2.81E−01	0.7

C=O is one of the most active functional groups during low-temperature oxidation of coal^29^. In the low-temperature oxidation process, aliphatic hydrocarbons are easily attacked by oxygen to generate C=O, which can be further oxidized and decomposed into CO, CO_2_ and other gases. It can be seen from Table 4 that after the adiabatic oxidation experiment, C=O in high-sulfur electrolytic coal H_2_ is 3.2 times that of raw coal H_0_, while H_3_ and H_4_ are 10.6 and 9.3 times that of raw coal H_0_. This shows that the electrochemical oxidation form and product of pyrite can promote the formation of C=O in coal during low-temperature oxidation. The content of C=O in H_4_ is lower than that of H_3_, because Fe^3+^ has a stronger ability to catalyze coal oxidation than Fe^2+^. Fe^3+^ promotes the further oxidation of C=O in coal to generate CO and other gases. This is the reason for the lowest temperature and the largest generation of CO gas in H_4_ in the adiabatic oxidation experiment.

Aliphatic hydrocarbon structures –CH_3_ and –CH_2_– are active groups in the process of coal low-temperature oxidation^30^. This paper selects the –CH_2_– antisymmetric vibration absorption peak near 2923 cm^−1^ with obvious changes to analyze the content changes of aliphatic hydrocarbon structure in coal. It can be calculated that the –CH_2_– content in high-sulfur electrolytic coal H_2_ is 1.2 times that of raw coal, indicating that electrochemical oxidation of pyrite can promote the development of aliphatic hydrocarbons in coal. The content of –CH_2_– in H_3_ and H_4_ is 0.9 and 0.7 times that of raw coal H_0_, indicating that iron ions promote the oxidation of aliphatic hydrocarbons. Comparing the changes of –CH_2_– content in H_3_ and H_4_, it is found that Fe^3+^ has a stronger catalytic effect on aliphatic hydrocarbons in coal than Fe^2+^, and is positively correlated with the catalytic C=O oxidation.

It can be seen from Fig. 4b that after adiabatic oxidation of the coal sample, the content of –OH in the H_2_, H_3_ and H_4_ of the coal sample is significantly reduced. Among the five types of –OH, free –OH, OH–OH, OH–π, OH–O and Aromatic ring –OH, the OH–OH hydrogen bond absorption peak near 3410 cm^−1^ has the highest intensity. Under low-temperature oxidation conditions, the content of OH–OH hydrogen bonds in coal is greatly reduced. The peak fitting results show that the OH–OH in H_2_ is 0.8 times that of raw coal, and after Fe^2+^ and Fe^3+^ are added to electrolytic coal, the OH–OH hydrogen bonds in H_3_ and H_4_ are 0.8 and 0.7 times that of raw coal. It shows that the catalytic effect of electrochemical oxidation on –OH oxidation is stronger than that of Fe ions on –OH oxidation.

### Mechanism analysis

**Figure 6:**
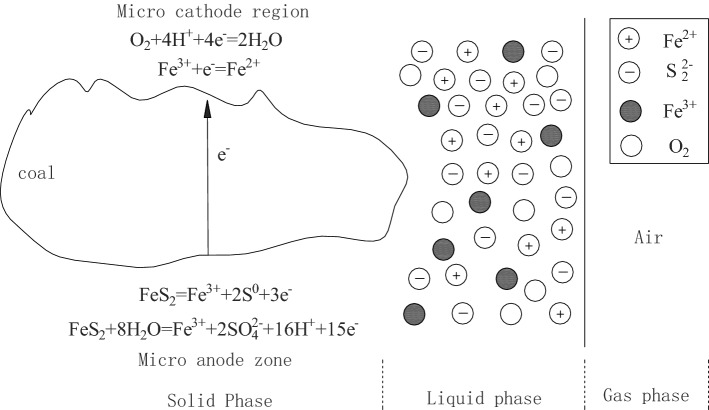
Electrochemical oxidation of pyrite in coal.

Comparing the characteristic temperature, heat release, self-heating capacity and CO generation characteristics of low-sulfur coal after electrolysis, it can be obtained that the electrochemical oxidation of pyrite can promote the spontaneous combustion of coal. FTIR analysis shows that the electrochemical oxidation of pyrite has a great influence on the active groups in coal. Yangquan anthracite coal is a highly metamorphic coal, and its electrical properties are semiconductors. Its electrical conductivity is the strongest among coals, and the carriers are mainly electrons. Pyrite undergoes electrochemical reactions in coal as shown in Fig. 6 When the electrochemical oxidation of pyrite continues to apply an external electric field to the coal, the coal body appears polarized. Polar groups such as –OH, –NH_3_, phenol, and ether C–O in coal gradually turn to the opposite direction of the external electric field under the action of the external electric field, which increases the surface active groups of the coal. This causes the formation of space charges in the coal, resulting in increased polarity of the coal body, so that the coal surface active groups, functional groups, and bridge bonds are in an excited state that is easy to be oxidized. This makes the coal oxidation require less energy. –OH, as an electronegative group in coal, is affected by external electric field force, which makes its oxidation activity maximum during low-temperature oxidation, and its content changes the most. This can explain the largest change in –OH content of high-sulfur electrolytic coal during low-temperature oxidation. Secondly, under the action of an external electric field, the random free electrons in the coal are aligned, and the trapped electrons in the groups are transformed into free excited electrons, which makes the free radicals increase significantly^17^.

Thermodynamic analysis shows that Fe^2+^ and Fe^3+^ have a strong catalytic effect on low-sulfur coal low-temperature oxidation. This is because Fe^3+^ and Fe^2+^ act as free radicals to catalyze the breaking of R–H and O–H in the structure of hydrocarbons and alcohols to generate R and RO, accelerating the oxidation of coal. Analysis shows that Fe^3+^ after electrochemical oxidation promotes the low-temperature oxidation of high-sulfur coal stronger than low-sulfur coal. This shows that the form of electrochemical oxidation has a further promoting effect on iron ions catalyzing coal oxidation. The two can be coupled to catalyze the spontaneous combustion of coal. This is because the polar groups in coal turn to the cathode area under the action of an electric field, and the oxidation reaction conditions provided by the cathode environment and the presence of iron ions can better promote the reaction of the active groups.

## Conclusion

1. In this paper, electrolysis is used to promote the electrochemical oxidation of pyrite in Yangquan high-sulfur coal. The shortest ignition time of high and low-sulfur coal samples after 8 h electrolysis is 0.86 and 0.98 times that of raw coal. It shows that the oxidation form of pyrite promotes the natural oxidation of coal.
2. Perform thermodynamic experiments on high-sulfur and low-sulfur electrolyzed coal with Fe^2+^ and Fe^3+^, and it is found that iron ions have a stronger catalytic effect on high-sulfur coal. The electrochemical oxidation form of pyrite and its oxidation products have a coupled catalytic effect on coal spontaneous combustion.
3. Combined with FTIR test and analysis, it is found that the electrochemical oxidation of pyrite causes the spatial polarization of Yangquan anthracite, and promotes the more active polar groups in the coal to be in an excited state that is prone to oxidation reactions. Due to the high content of -OH in Yangquan high-sulfur coal, electrochemical oxidation has a greater impact on -OH oxidation. Iron ions have a stronger catalytic effect on aliphatic hydrocarbons and promote the reaction of coal to form CO under low temperature conditions.
